# Physicians’ professionalism from the patients’ perspective: a qualitative study at a single-family practice in Saudi Arabia

**DOI:** 10.1186/s12910-023-00918-9

**Published:** 2023-06-07

**Authors:** Eiad AlFaris, Farhana Irfan, Noura Abouammoh, Nasriah Zakaria, Abdullah MA Ahmed, Omar Kasule, Dina M Aldosari, Nora A AlSahli, Mohammed Ghatar Alshibani, Gominda Ponnamperuma

**Affiliations:** 1 Department of Family and Community Medicine, College of Medicine, King Saud University Chair for Medical Education Research and Development, King Saud University, Riyadh, Saudi Arabia; 2 Ehealth Unit, Faculty of Medicine, University Malaya, Kuala Lumpur, Malaysia; 3 Academic and Training Affairs, King Fahad Medical City, Riyadh, Saudi Arabia; 4 King Khalid Hospital, King Saud University, Riyadh, Saudi Arabia; 5 Department of Family and Community Medicine, College of Medicine, King Saud University, Riyadh, Saudi Arabia; 6 Department of Medical Education, Faculty of Medicine, University of Colombo, Colombo, Sri Lanka; 7 College of applied science, Al maarefa university Riyadh Saudi Arabia, Riyadh, Saudi Arabia

**Keywords:** Physician professionalism, Patient perceptions, Patient satisfaction, Qualitative study

## Abstract

**Introduction:**

Professionalism is a crucial component of medical practice. It is a culturally sensitive notion that generally consists of behaviors, values, communication, and relationships. This study is a qualitative study exploring physician professionalism from the patients’ perspective.

**Methods:**

Focus group discussions with patients attending a family medicine center attached to a tertiary care hospital were carried out using the four gates model of Arabian medical professionalism that is appropriate to Arab culture. Discussions with patients were recorded and transcribed. Data were thematically analyzed using NVivo software.

**Results:**

Three main themes emerged from the data. (1) In dealing with patients, participants expected respect but understood delays in seeing physicians due to their busy schedules. In communication, participants expected to be informed about their health conditions and to have their questions answered. (2) In dealing with tasks, participants expected proper examination and transparency of diagnosis, but some expected the physician to know everything and did not appreciate them seeking outside opinions. They expected to see the same physician at every visit. (3) In physician characteristics preferences, participants preferred friendly smiling physicians. Some cared about the outer appearance of the physician whereas others did not.

**Discussion/conclusions:**

The findings of the study explained only two themes of the four gates model namely dealing with patients and dealing with tasks. Cultural competence and how to benefit from patients’ perceptions to be an ideal physician should be incorporated into the process of physicians’ training.

**Supplementary Information:**

The online version contains supplementary material available at 10.1186/s12910-023-00918-9.

## Introduction

Professionalism has been recognized for many years as a crucial component of medical practice and medical education [1–5]. Medical professionalism has received increased attention in medical education recently as part of physician competence [6] and is considered an important outcome in Saudi Meds (a competence specification for Saudi medical graduates) [7]. Novel approaches including visual aids are being used to teach professionalism because of its importance [3]. In teaching professionalism, particularly for medical students and residents, the use of case vignettes is recommended because it boosts student centered learning, integrity, feeling empathetic towards patients, and facilitating collaboration through reflection [8].

At the undergraduate level, professionalism is taught and learned mainly through small group learning discussions at a formal level and through role modelling at an informal level. At the postgraduate level, professionalism is taught and learned through feedback received mainly for workplace-based learning. At the level of continuing professional development, learning professionalism would mainly happen through peer influence and discussions. At all three levels, the role that reflective practice plays, is undisputed and of paramount importance. Furthermore, the role of reflective practice assumes a larger proportion as one moves from undergraduate through postgraduate to continuing professional development [9].

Professionalism is a moral phenomenon and is a cornerstone of the physician–patient relationship as well as medicine’s relationship to society, also known as a “social contract” [10]. Professionalism has no simple overall definition but can be defined for each individual discipline [11, 12]. In general, however, it includes behaviours, values and relationships required by the medical profession while serving the patients and the society and underpins the trust the public has in doctors [13]. Medical professionalism is a set of attributes to be mastered by healthcare professionals and is a critically important competency that is complex and hard to assess [14]. Studies from different parts of the world have shown that the attributes of professionalism, important from a patient’s perspective, are the doctor-patient relationship [15], patient trust in the physician [16], competence, respect, communication skills including interaction with the Internet [17], and integrity [18].

Attempts have been made to define dimensions of professionalism [19, 20] but there is no unanimity. Few studies have attempted to define the domains of professionalism as attitudes and behaviours using quantitative and qualitative methods. Wagner et al. identified knowledge/technical skills, patient relationship and character virtues as main themes of professionalism [21], while Jha et al. have identified compliance to values, patient access, doctor-patient relationship, demeanor, professional management, personal awareness, and motivation as the themes of professionalism [22]. The emotional intelligence of the physician is recognized as important in physician-patient relations [23] and should be considered in professionalism.

The issue of patients’ perceptions of healthcare professionalism is important for both researchers and organizational structures. Medical professionalism has been shown to affect doctors’ relationships with their patients, quality of care, and ultimately health and illness outcomes. Patients look for doctors in charge to have a high standard of professional behaviour. Patients perceive communication skills and compassion as an important aspect of physician professionalism [24].

The cultural background of Arab countries is different from Western countries [25]. Therefore, Western frameworks of medical professionalism may not resonate with the cultural values of Arab countries. Arabs’ professionalism values are not necessarily from an Islamic perspective but rather from some common values that are followed by the natives of these countries. The four gates were identified from a Delphi study in one study [26]. The themes in this study were developed to formulate a professionalism framework for healthcare providers as interpreted by local medical professionals in Arab countries. The framework consists of (i) dealing with self, (ii) dealing with tasks, (iii) dealing with others and (iv) dealing with God. Dealing with self includes self-awareness, balance between personal and professional roles (clinicians, teachers, scholars and community leaders). Dealing with tasks includes excellence and commitment to professional development. Dealing with others includes respect for patients, colleagues and students and keeping professional confidentiality. Dealing with God includes self-accountability for own behaviours and self-motivation. Cultural differences sometimes lead to different professional attitudes and roles for example, the doctor maybe looked at like any other person regarding culture-based attitudes. For example, as the norm in the Arab society for the women to avoid eye contact with a man, the same phenomenon may take place with the patient visiting a male doctor [27]. Religious and cultural imperatives allow a paternalistic model of patient care to be the norm. This society relies heavily on the role of the male or the family in decision making [24–26]. Saudi patients in general prefer their male physicians to wear the Saudi national dress when discussing their psychosocial issues [28], this could be attributed to cultural similarities.

In the present era, an awareness of national and cultural differences is necessary when both ideas and individuals appear to be changing. It is important to determine, from the patient perspective, the elements of medical professionalism that are critical to patients and care givers. In doing so, we explore medical professionalism in Saudi Arabia and provide recommendations on medical professionalism through the medical education system.

Many studies have observed professional attitudes among doctors, but notably, there is little research that has explicitly explored patients’ perspective, particularly in the domain of professionalism in an Arab context. Professionalism has been identified as one of the most important competencies to be mastered by healthcare professionals. Patients’ perceptions of their physicians’ competence and knowledge and their confidence in the physician can reduce patients’ anxiety and its detrimental effects on the outcome of care [13]. There is evidence that physicians’ conduct relates directly to overall patient satisfaction with health care services [29]. A systematic literature review on the satisfaction-measurement instruments suggests that there is no gold standard instrument for the measurement of user satisfaction in health care services. Furthermore, the concept of satisfaction may differ across cultures. Patient satisfaction construct needs to be assessed using a multidimensional approach [30]. Therefore, the current study aims to assess the patients’ perceptions of physicians’ professionalism using a qualitative approach to be able to collect non-numerical data to gain insights. understand and explore what constitute professionalism to patients.

This study aimed at exploring the research question: what are patients’ perceptions towards physicians’ professional behavior?

## Methodology

### Study design

The current study is a phenomenological study eliciting patients’ views and experiences of medical professionalism using focus group discussions (FGDs). The benefit of this methodology is that it creates the best opportunity to ‘give voice’ to the experiences of patients. Phenomenological approach was used as it provides a rich description of the lived experiences of the participants [31]. Focus group discussion was used for this study as it provides diversity and enrichment of experiences from a wide variety of participants’ profiles. It facilitates a better way for exchanging viewpoints and discussing disagreements between participants that cannot be captured in a one-to-one interview.

### Study setting and target population

The population targeted was Saudi patients attending primary care clinics (PCCs) in King Saud University Medical City (KSUMC). The PCCs of KSUMC are part of the Family and Community Medicine Department.

### Sampling and recruitment

Principles of purposive criterion sampling strategy was adopted. In addition to willingness to participate, participants who had the knowledge and experience of encountering physicians for medical purposes were invited. A convenient sample of 6 to 8 patients per focus group [31] were selected. Only participants who visited the clinic were selected as the data needed are related to the patients involved in the medical encounter. The groups were gender homogenous as is the cultural norm in Saudi Arabia. The total sample size was determined by the principles of data saturation.

Researchers identified the sample from clinic patient lists, one or two days before the clinic visit, and approached the patients by telephone to provide information about the research topic, its aim and what was required from the participants and to seek initial verbal consent to participation in the study. Once they arrived at the clinic, they were contacted in the reception area. They were asked if they needed clarifications or if they had further questions. After that a written consent was obtained. Patients who fulfilled the criteria of physical fitness and time availability were included in the study.

### Data collection

The main focus and structure of the interview guide was based completely on the four gates framework [26] as it applies to the Arabian context. The other two frameworks namely the Royal College of Physicians (RCP) and the American Board of Internal Medicine (ABIM) professionalism framework were used to obtain examples and explanations that helped us in the design of the interview guide (Appendix 1). Some of the values and behaviours mentioned in the frameworks may not be reflected by patients as they are unlikely to be observed such as, dealing with self and dealing with God. Patients were encouraged to narrate their lived experiences with physicians and to reflect on their overall experience rather than only on the current visit.

It was made clear to the patients that their participation was voluntary and that withdrawal from the study was allowed at any time without any negative implications on the health services provided. They were assured that their information would be anonymous and would be kept confidential.

Each FGD was recorded using a small digital recorder. Consent for voice recording was requested before the start of the discussion. Participants were told that they had the right to ask the researcher to stop recording at any time during the discussion. Audio recordings transcription of the discussions were completed no more than a week after the discussion and were destroyed later on. To allow data from each discussion to feed into the subsequent one, analysis of each transcript was completed before the subsequent discussion. Transcripts were saved to a file of one of the authors and secured with a password. Demographic information was collected at the start of the discussions. The qualitative research was used to reflect the true picture as it deals with reflecting thoughts and experiences. All ranges of different views and perceptions that emerged from the data were documented with a note that, one participant, few or most of the participants shared a specific theme.

The FGDs were conducted in the seminar room of the PCCs. Each FGD lasted about an hour. All FGDs were conducted by the same two researchers [FI, NA] and a moderator, third researcher was included to manage late arrivals and take notes. Both interviewers were not involved in the treatment or clinical care of any patient which guards against potential power dynamic conflict. A topic guide was used throughout the sessions to guarantee consistency of questions across all FGDs with the flexibility to add more questions to consequent discussions as per patients’ responses. Data collection continued until data saturation was achieved.

### Data analysis

The directive content analysis method of qualitative data was used to assess the attributes of medical professionals, whereby the attributes of medical professionalism were derived from an existing framework (pre-set reference framework). The thematic analysis approach proposed by Ritchie and Spencer [32] was used. It provided a sequential structure for data analysis. The first step was familiarization by listening to the recordings; transcribing them verbatim; translating; reading and re-reading through the data to gain a holistic overview and be familiar with the range, depth, and diversity of the information. The second step was transcription and descriptive coding using the NVivo 10 qualitative data analysis software. All researchers read the same transcripts and agreed on an initial coding frame that was applied to subsequent transcripts with flexibility to add more codes. The third step was basic analysis to identify emerging themes and subthemes. The codes were grouped logically to answer the research question. Data were rearranged according to their thematic orientation. The fourth step was interpretation. Priori themes and concepts were arranged as per the Four Gate model adopted and some other newly emerging themes were detected from the analysis. Subthemes were extracted directly from the data. Key themes were developed, and quotations were used as supporting evidence. The thematic network was created to map the nature and range of phenomena as well as associations between themes. These processes continued until theoretical saturation. The participants were not given feedback on the transcripts and data findings.

## Results

Fourteen patients participated in the three focus groups (Table 1). The results were categorized according to participants’ responses and three main themes emerged from them: dealing with patients, dealing with tasks and patient preferences and concerns.

**Table 1:** Participant socio-demographic particulars

Participant	Sex	Age range	Times visiting the doctor/year
**1**	**Male**	**65–75**	**4–5**
**2**	**Male**	**45–55**	**5**
**3**	**Male**	**35–45**	**12**
**4**	**Male**	**50–60**	**4–5**
**5**	**Male**	**20–30**	**5–6 (with his father)**
**6**	**Female**	**75–85**	**4**
**7**	**Female**	**50–60**	**7**
**8**	**Female**	**45–55**	**6**
**9**	**Female**	**15–25**	**3–4**
**10**	**Female**	**40–50**	**4**
**11**	**Female**	**60–70**	**6**
**12**	**Female**	**30–40**	**5**
**13**	**Female**	**35–45**	**6**
**14**	**Female**	**65–75**	**6**

While one theme emerged inductively, two themes emerged deductively from the data and were revealed from the Four Gates Model.

## 1. Dealing with patients

### (A) Respect for patients

#### Care and good relationship

Older participants expressed their desire to be welcome by the physician and be given status for their age. According to them, this will make the patient more comfortable and keener to interact with the physician: ***“****He (the doctor) gives him (the old patient) a value, for example. This makes the patient feel comfortable and interact effectively with the doctor” P1*.

The participants described consequence of not being welcome by their physician: *“it becomes a formal doctor-patient relationship. So, I answer his questions only… The doctor sees the lab results in front of him in the patient’s history file, and the patient responds to questions only.” P4*.

However, the same patient had another experience with another physician when he felt that the latter was more caring: *“Once, the doctor, helped me taking off my socks in order to see my toes and see if there were wounds because I am diabetic” P4*.

In general, most participants described their relationship with their physicians as “good” despite describing it as “checking files and assurance” with minimum conversation.

### Appreciating patients’ time

It seemed that all participants in all the groups did not blame physicians for their long waiting time and they did not expect them to apologize or show any kind of appreciation of patients’ time. One patient reported: *“A doctor rarely comes late. You can’t tell her/ him you’re late because the delay is due to the procedures that precede seeing the doctor. You need to wait in the reception (room) because of the high number of appointments. But when you see the doctor the reception from him is better” P1*.

The participants showed their appreciation of physicians’ busy schedules as shown below: *“If the doctor was free, he will surely respond to you…(for questions related to medicines)…they have many patients.“ P1*.

Participants seemed to blame the appointment booking system for delaying consultations. Some other participants believed that physicians do not use the allocated appointment time appropriately. One mentioned: *“Of course, the appointment doesn’t take 15 minutes, maybe five minutes: …Sometimes the doctor utilizes the ten minutes left from my time to see another patient … because more appointments have been imposed on him.“ P5*.

Participants added that some physicians do apologize for the patients waiting time although patients do not usually blame them for that delay: *“Sometimes you’ll meet doctors who will apologize and explain what happens. So, you’ll feel comfortable seeing them, but sometimes if he just apologizes to you, your anger fade even if you wanted to express your anger about it " P4. “When we see the doctor, it is a five-star service frankly.” P8*.

### Lack of respect

A small number of the participants shared stories in which they felt looked down upon by their physicians. For example, one participant was accused by her husband’s doctors of not taking her husband’s health condition seriously: *‘He made me feel that he is the only one who is discerning and the world is nothing. He was talking to me with superiority.“ P7*.

On the other hand, one of the participants was appreciative of the way her son’s physician approached her and talked to her. She noted: *“My son was diagnosed with liver failure. I swear, the doctor was sitting with me and asked me to be patient and told me it is God’s fate and they can do something about it. I pray for this person everyday. I pray for him all these 12 years” P8*.

### (B) Communication with patients

#### Informing and explaining

Although a small number of the participants did not need information about the medication prescribed, most of the young and older participants expressed their need for proper communication regarding medications. They knew the times of taking the medications and the number of pills they need to take. However, they expressed their need to know the uses of the medications they consume. Especially after changing the company of the medication. For example, *“He (the doctor) writes the treatment for you, but he doesn’t explain to you about it or its effects. Therefore, you go back home and don’t know what this medicine is for.“ P2*.

When the patient was asked about whether he dispensed the prescribed medication, he replied:“ *No, I went to a private clinic at my expense … I took another treatment*.“ P2.

Although, all participants acknowledged that if they asked the physician or the pharmacist about the uses and side effects of the medications, they would reply.

#### Answering questions

Participants also raised another point regarding asking questions. Older participants thought that there was no need to ask questions and they gave excuses for physicians not explicitly allowing them to ask questions: *" I don’t need to ask questions, if there is something, the doctor will tell me.“ P3*.

However, they appreciated the good experience in that regard: *“Once the doctor said, “Would you like to ask about something?“ I thanked him for that and appreciated it” P3.*

Some other participants showed their frustration with physicians who did not give them a chance to ask questions: ***“****He (the doctor) says goodbye to you meaning that you need to leave the office now. If you wanted to ask a question, you hesitate” P2.*

Fear of asking questions during the consultation was also mentioned by some participants: “*We are scared to ask or upset the doctor because we need him… I don’t want to cause any trouble between me and him because he is treating me” P6.*

#### Casual communication

All participants agreed on patients’ need to be cared for by their physicians. This care can be manifested by casual questions about health and family or conversations about football matches. For example, one participant noted: ***“****These conversations starting with, how are you, how are things? what’s the news? the medicines are good or not? This can add more comfort to the patient than when he hears, you are good and stable. Sometimes my doctors talk about football match or anything else…it comforts me” P4.*

## 2. Dealing with tasks

Experiences of the participants included both positive and negative ones. The following explains their experiences and shows their views about them.

### (A) Examination

The patients expressed their frustration with physicians who do not do proper physical examination. One noted: *“I had a situation in the orthopedic department about two weeks ago. I suffer from knee pain. I entered the doctor’s office but the doctor didn’t even get up from his chair. He didn’t touch my knee; he didn’t even get close to it. He said to his assistant “Write the treatment…write to him… write to him, write to him,“ and that’s it. I wondered how he became a doctor.“ P3.*

### (B) Making a diagnosis

Participants opinions on physicians’ transparency about needing time to consult others about the patient’s condition varied. While some felt that this attitude is acceptable to get a proper diagnosis and expressed their willingness to visit the same physician again, *“He (the doctor) says, we’ll see when the tests come out. We’ll see…of course he gives himself a chance to review the case. He makes me feel that he knows everything about my case but in fact he doesn’t.“ P8*.

Others thought,*“ If he (the doctor) told you that he needs time to consult a colleague or read about the disease, you would feel stressed. He should know about his specialty. He is supposed to be experienced in the field. " P10.*

### (C) Continuity of care

It seemed that patients did not like being seen by a different physician from the follow-up team during their visit. They had different views about visiting the initial physician and seeing the “alternative” physician. Alternative physician usually includes residents and fellows working in the same team with the main assigned physicians. According to them, the alternative doctor just writes the prescription based on the primary physician’s previous notes and prescriptions. *“The primary physician discusses the issues. He explains to you about what medication has been added and what has been reduced. He asks about your feelings. He changes the dose if needed, but you feel that the alternative doctor just refills the medication” P2.*

All participants complained from not being informed about the availability of their physicians, so they end up seeing another one: ***“****After every five appointments, I see the primary doctor once. You don’t know where he is, on vacation, present or not… No one tells you” P10.*

Another participant highlighted patient’s need to see the same physician, he noted:

*‘It is better to keep going to the same doctor than to go to a different one, who you don’t know, each time. Your usual doctor will understand your condition and recognizes you. You will feel comfortable with him. " P3.*

Another one added: *“It depends on the patient’s psychological comfort, 50% of the treatment for the patient depends on his trust in his doctor” P9.*

## 3. Patient choices about physician characteristics

It seems that patients do develop an idea about physicians as soon as they see them in the clinic. This idea contributes to their judgement on whether to trust thee physicians. These included:

### (A) Age of the physician

Ideas about the influence of the age of the physicians on patients’ overall decision about their competence varied. One participant noted: *“When I see a doctor for the first time, I wonder about his age, whether old or young. The doctor’s age tells you whether he has long experience, so you trust him.“ P2.* At the same time, some other participants who held the same view, commented:*“But the one who asked me about my condition more and showed enthusiasm was a young doctor… An expert doctor may have more knowledge” P4.***“***I like an expert doctor. Even if I need to search for him, I will prefer to go to an expert doctor. I like expert doctors. Experience plays a role. I like older doctors*” P8.

Another female participant felt more comfortable dealing with younger physicians even if they are less experienced: *“I noticed that young doctors are more understanding. They live in a time where everything is more developed, unlike the old. … when one of them would talk to me, for example, she or he would call me (my mother or my sister) However, the old are more formal. At the end, a comfortable relationship is better than a formal one.” P10.*

### (B) Gender of the physician

Although a female participant explained the cultural background for local patients preferring male physicians, male participants thought that they may prefer male physicians for other reasons: *“Since we are a patriarchal society, there is a stereotype that male doctors are more skilled and experienced than female ones. It is possible, however that a woman may be smarter than a man and may have capabilities which are better than his, but the common stereotype about men is what makes people think the opposite. Even if a female doctor is competent, people’s internal feeling would go for a male doctor.“ P8.*

Nonetheless, a male participant expressed: *“There are some private questions that are difficult for you to ask to female doctor” P5.*

Another male participant had a contrary view: *“I prefer female doctors. They are more gentle and kinder. Their hands are lighter, especially in ophthalmology and dentistry. They are more accurate and skilled.“ P3.*

### Friendliness and outer look

Participants showed their preference for naturally smiling physicians however, “good” physicians were overall preferred: ***“****There are some persons who don’t prefer to smile but they are good doctors. Others are mostly smiley. If he is a good doctor, then this thing is the most important to me.“ P8.*

The same view was held about physicians’ outer look, one participant expressed it as follows: *“Most important, the doctor should be knowledgeable. I like doctors with neat appearance. Not only doctors, I think all people should be neat …For me, the most important thing is a doctor’s competence and knowledge only.“ P10.*

Another participant added that confidence in female physicians can be lost if they are overdressed: “*We, especially women, care about our appearance too much. One may go to work wearing Versace’s shoes or Christian Dior’s perfume. It’s too much. You’re in a workplace. I feel they care about their appearance more than their work.“* P7.

Some participants had great experience with the physicians. One participant mentioned “*My husband was admitted in the hospital. He caused the staff a lot of trouble. They were very patient with him until they were able to help him. They used to come to him every day as if they were his daughters and sons. They didn’t behave as doctors, and he would wait just to see them.“ P7.*

## Discussion

Three broad themes emerged to classify the responses on medical professionalism. Participants tended to categorize their responses either to the patient-centered elements or physician’s competencies. These themes, however, could not fully explain or entirely address the four parts of the model. Hence, much of the domains that emerged are in keeping with a patient centered approach and looking at patients’ preferences and concerns regarding this approach. The results supported the two domains of medical professionalism derived from the four gates model [26], namely dealing with others/patients, dealing with tasks. This indicates that dealing with God and dealing with self are personal attributes. As this study was with patients, it was impossible for patients to comment on this as this is something that only the physicians would know, i.e., personal information about physicians.

Determining patient experience has become a foremost aspect of quality improvement in healthcare. Primary care services are the first contact of the patients and therefore, patients’ perceptions and their prioritizing aspects of the key attributes of doctors’ professionalism is of prime importance. Studies have demonstrated that perspectives may vary; as socio-demographic variables can influence patients’ expectations, satisfaction, and perception of the quality of medical care [33–35].

### Dealing with patients

The doctor–patient relationship was considered a prime element of the consultation. General features included respect, care and communication between both parties. Results from Wiggins and colleagues demonstrates that patients highly rank physicians’ behaviors related to communications skills [36].

All participants in the current study agreed that patients need to be cared humanely and friendlily by the physicians. Physician presence of mind is an important aspect of this relation [37]. A caring and respectful physicians’ attitude was valued highly by patients for satisfaction. This goes in line with the results of a large data set from the US Consumer Assessment of Healthcare Providers and Systems (CG-CAHPS), where interpersonal aspects of care were key determinants of patient satisfaction, and the most important predictor of patients’ overall physician rating was whether the physician showed respect [38]. Another study on physician professionalism from a generation perspective has shown that respect for patient, compassionate patient care and kindness, respectively, were the most important values for physicians [39]. This emphasizes that each person is unique and should be treated appropriately and differently as patients’ personal experiences change their perspectives of physician professionalism. Respect is a multi-faceted and personalized task. For some, it may be just expressions of care while for others it might be help, listening and good relationships. Patients are generally aware of the degree to which their doctors respect them [40]. Consistent with the previous research, the findings of the study suggest that elements of care, empathy, good relationship, and attention to needs may be important components of what it means to respect patients as individuals [41, 42]. Patients described these elements as ways to recognize persons as valuable and physician’s respectful attitudes were considered important in improving communication. Under this domain, another issue highlighted was effective communication with the patient. This indicates a conceptual shift in medical care with emphasis on patient autonomy [43].

In doctor-patient relationship, the communication of expert knowledge and emotions is central. However, it is likely that the verbal and non-verbal gestures of physicians such as smiling, leaning forward or rapport building in the form of casual statements along with doctors’ ‘humaneness’ (warmth), giving sufficient information and time are important aspects too [44, 45].

Physicians can enhance patient satisfaction by allocating a segment of consultation for chatting about nonmedical topics, and by allowing time for exchange of views with the patient [46]. Our study suggests that physicians need to be aware of their attitudes towards patients, as their feelings might affect their actions and thus be recognized by patients.

### Dealing with tasks

The traditional paternalistic model of medical decision-making has become outdated. Patients prefer active involvement and continuity in their care. Some of the general expectations that seemed especially important to patients in this study included: physicians’ competence, patients’ involvement, and the continuity of care. Physician competence is a priority and plays a major role in patient satisfaction; whether it is examination, medication prescribing, or physician’s communication skills/ competence provides comfort and reassurance to the patient that they are in safe hands. It is a sign that all the possible actions of treatment have been explored [47]. Effective communication, whether it be sharing of information or a common understanding during the consultation could help to develop swift trust [48]. A study on doctor-patient communication on drugs has shown that the patient involvement and sharing the information of medications were not generally present in the consultations [49].

Furthermore, patients in this study reflected on the importance of continuity of care. Consulting a physician often, can be explained by game theory [50], which indicates a history of past interactions between a doctor and patient; anticipation of future interactions makes cooperation and good quality care more likely. We found examples of patients who were uncomfortable when consulting an unfamiliar physician. They were more skeptical and disappointed. Continuity of care signals trustworthiness; is an important aspect of health professional-patient interactions; which is linked with outcomes like patient satisfaction, adherence to treatment or advice [48].

Establishing a good relationship is critical both for physicians and patients. As evidenced by the narratives in this study, the nature of this relationship among individuals varies as patients recognize physicians’ competencies. Our findings suggest that only longevity of consultations over time is not enough to develop trust. Though some degree of continuity is needed [51], trust is also reliant on what happens during the consultation. More investigation is needed to determine what drives patient preferences and how best to incorporate this into communication education and techniques for providers.

### Patient’s preferences/concerns on physician characteristics

Medical professionalism perception is affected by individuals’ personal backgrounds, cultures, socioeconomic status and age The values differ among each generation of individuals. Healthcare providers as well as patients come from different generations, these generational differences can give rise to difference of opinion in the professional values/acts [39, 52].

Our study has also identified a new domain not present in the original four gate model. Under this domain, patient preferences were addressed related to the physicians’ personal attributes like age, gender, manners etc. were addressed. To date, the literature on the information on patient choice of physician with specific attributes has been very limited.

Age was an important factor for patients. Patients in this study showed a preference towards elderly physicians with a view that they will be more experienced. These findings are in line with those of other researchers [53]. Many other factors might also have an effect on the patient interaction including but not limited to gender, attitudes, and cultural differences [46]. There are differences in the way patients prefer their physicians keeping in mind the culture and religion. These differences include privacy, touch restriction, and patriarchal values that are inherent [54].

Our findings agree with international and national studies that showed patients’ preferences for a clinician of the same gender as it gives a comfortable feeling to discuss their health problems with physicians of a similar sex particularly if they require a physical exam [55–58]. The nature of Arab society may influence the patient’s decision when choosing a treating physician, whether male or female, gender plays an important consideration. Although some patients who preferred female physicians in certain specialities like ophthalmology. Another one was the preference to see a female doctor as she gave him the right diagnosis! This was not reflected in the findings.

Non-verbal communication was recognized in this study as being important throughout the medical interview and an important factor in doctor-patient interactions [59]. An observational study from Poland, showed that doctors’ tone of voice was associated with patients picking up signs that the doctor is not interested in them [60]. Furthermore, study has shown that a friendly manner with a smiling face and semi-formal dressing was preferable by patients than a flashier sartorial style [61].

Although more work needs to be done to further explore this area, there is significant evidence that considerable attention needs to be paid by physicians to check their own non-verbal behaviors [44, 62, 63]. From the psychodynamic perspective, an understanding and insight towards patient’s psychology is of paramount importance for satisfaction.

Being respectful means that the physician should not impose his/her own values because of being expert. The benefits of respecting patients are considerable. Respect generates trust, and makes it easier to work together as partners.

In assessing patients’ perceptions of their physicians, it was found in the current study that they have the desire that doctors respect them in the form of care, welcoming and spending enough time in the consultation. Furthermore, good communication skills included explaining ailments and drugs use; listening carefully; providing easy to understand instructions and casual questions about different life issues. We recognize the immense time pressures physicians operate under and the many competing demands on their time. Their cognitive capacity and emotional resilience in the face of obstacles and frequent disappointments are very important. But the increasing call for meaningful patient and family engagement in healthcare, driven in part by a growing dissatisfaction with a profession that is often perceived as unwilling to share information and slow to change, requires some introspection.

### Strengths and Limitations

This qualitative study has provided an insight into the patient’s views on the medical professionalism in an Arabian context. This study is one of the first to explore this area from Arab patients’ perspective to generate valuable explanatory insights into mechanisms underlying observed relationships. Our findings are context-specific to patients in Saudi Arabia so readers should interpret our findings accordingly and against our description of the study context when considering transferability to their own patient groups and institutions.

The findings of this study may not be generalized to other communities. They represent the participants visiting a single-family practice and it highlights the general view of professionalism among Saudi family practice attendees. The generalizability of the findings is a significant concern as the sample size was too small and the setting did not represent the whole country.

Further multicentric studies using a mixed methodology is needed to make the results generalizable. All areas of four gate model could not be covered due to peculiarity of the model from a physicians’ perspective. This study is one of the first to explore this area from Arab patients’ perspective to generate valuable explanatory insights into mechanisms underlying observed relationships. It should be noted that the perception and experiences of patients who are involved in the medical encounter in a family practice was included and this may exclude those who prefer not to visit the clinic for issues related to physicians’ professionalism.

For each FGD, participants’ feedback was achieved through utilizing techniques such as paraphrasing and summarization for clarification. However, the study might benefit from respondent validation at the level of the study findings.

For each FGD, participants’ feedback was achieved through utilizing techniques such as paraphrasing and summarization for clarification. However, the study might benefit from respondent validation at the level of the study findings.

## Conclusion

The findings of the study explained only two themes of the four gates model namely dealing with patients and dealing with tasks. The third, fresh theme found in this study was on patient preferences and concerns. We found patients’ concerns to revolve around physicians’ competence, behaviour, patients’ involvement, and the continuity of care. Of particular concern are the less favorable narratives given by patients for an uncomfortable environment in the consultations, whether it be because of physician’s experience, age, time constraint or nonverbal cues. Based on the findings, it is suggested to introduce into the curriculum of physicians’ training, how to incorporate patients’ perceptions towards being an ideal physician and being culturally competent. As patient satisfaction with the care provided is one of the aims of any healthcare system, future research may consider including these areas of concern for better assessment of medical professionalism from the patients’ perspective.

## Electronic supplementary material

Below is the link to the electronic supplementary material.

Additional File 1: The Focus Group Discussion (FGD) interview guide

## Data Availability

The datasets generated and/or analysed during the current study are not publicly available due to consent not being obtained from participants for this purpose, but are available from the corresponding author on reasonable request.

## Abbreviations

FGDs: Focus group discussions
PCCs: Primary Care Clinics
KSUMC: King Saud University Medical City
RCP: Royal College of Physicians
ABIM: American Board of Internal Medicine
