# Supplementary material for: Physicians’ professionalism from the patients’ perspective: a qualitative study at a single-family practice in Saudi Arabia
Source: BMC Med Ethics. 2023 Jun 7;24:39. doi: 10.1186/s12910-023-00918-9 (PMC10249207; doi:10.1186/s12910-023-00918-9)
Supplement: Supplementary file 1 — Additional File 1: The Focus Group Discussion (FGD) interview guide [file 12910_2023_918_MOESM1_ESM.docx]

**Appendix 1. The Focus Group Discussion (FGD) interview guide will incorporate the following questions:**

All interviewed focus groups were asked.

Can you tell me some information about yourself or your health condition? (Probe: for how long you had this condition?)

1. What do you understand by the term professional behavior in the context of medicine?
2. How do you describe your relationship with your doctors? Elaborate and narrate lived experiences please.

| **FOUR GATES** | **More details** | **Question in the FGD guide** |
| --- | --- | --- |
| Dealing with self | Self-awareness, recognize own potentials and limitations. | I am interested to hear your experience whereby your Dr(s) admitted uncertainty or ignorance on some medical information?  I am interested to hear your experience whereby your Dr(s), when running late or doing other pitfalls apologizes or does not |
|  | Self-management; balance between personal and professional roles | I am interested to hear your experience whereby your Dr was kind with himself and asks for help appropriately when exhausted or in difficulty.  **Are there more questions?** |
| Dealing with tasks | Excellence and commitment to professional development  Reflective practice | We are interested to know whether your Dr shares new and updated information and quotes some studies from the literature. |
|  |  | Q1. Can you describe an incidence when your doctor has asked your opinion and suggestions/feedback formally or informally on his/her performance and drawbacks?  Q2. Have you experienced a moment whereby your doc expressed his thinking process or insight or shared thinking loudly regarding a clinical, social, emotional, or behavioral situation? |
| Dealing with others | Respect for patients, colleagues and students  Keep professional confidentiality | Can you share with us any positive or negative experience(s) on how your doctor communicates with the nurses, trainees and others?  Can you narrate lived experiences with physician regarding his respect to you as a patient during the encounter?  **Probe:** i- discuss the trait of respect to his patients? Such as smiling, active listening (not interrupting, facilitation cues and summarizing), not rushing patients through. offering and discussing options with you and asking your opinion, passion and compassion.  **ii-** Treating you like you’re on the same level; never “talking down” to you or treating you like a child.  Letting you tell your story; asking thoughtful questions.  **iii**- Showing interest in you as a person; not acting bored or ignoring what you have to say.  **iv**- Encourage you to ask questions; answering them clearly; never avoiding your questions or lecturing you. |
|  |  | Can you share with us instances in which your Dr kept or otherwise your privacy and made sure that he keeps your information confidential. |
| Dealing with God | Self-accountability for own behaviors (taqwa 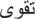)*  Self-motiva􏰃on: expect reward from God, not people (ehtesab )* | Can you share with us positive or negative experience on how your doctor worked for your benefit (even if not watched or rewarded) for example educating you about your health and safety? checking for drugs accuracy, Advising for the reputable specialist.  If yes tell us more and if no, we give Probes to facilitate the answers.  Do you feel that your doctor has Self-accountability for his or her behaviours even if not watched? Can you give me an example from your experience on this topic?  Do you feel that your doctor has Self- motivation or doing his tasks sincerely for Allah rewards not for external incentives? Can you give me an example from your experience on this topic? |

Other questions outside the four gates:

1. Does it matter to you if your doctor is male or female? Why? (Probe: Smiling, confident, physical appearance).
2. What other examples of good or bad doctors’ behavior that you can think of?
3. Is there anything else that you’d like to add to what you’ve already said?
